# Global Public Health Security

**DOI:** 10.3201/eid1013.070732

**Published:** 2007-10

**Authors:** Guénaël Rodier, Allison L. Greenspan, James M. Hughes, David L. Heymann

**Affiliations:** * World Health Organization, Geneva, Switzerland; † Emory University, Atlanta, Georgia, USA

**Keywords:** Health legislation, World Health Organization, public health surveillance, communicable disease control, public health, International Health Regulations, policy review

## Abstract

National public health institutes will play a key role in implementation of the revised International Health Regulations.

–—Dr. Margaret Chan, Director General, World Health Organization; World Health Day 2007

“When the world is collectively at risk, defense becomes a shared responsibility of all nations.”

The framework of the newly revised International Health Regulations is a key driver in the effort to strengthen global public health security. Unanimously agreed upon by the World Health Assembly on May 23, 2005, the regulations are the result of experience gained and lessons learned during the past 30 years. This global legal framework includes a commitment from the World Health Organization (WHO) and from each WHO member state to improve capacity for disease prevention, detection, and response. It provides standards for addressing national public health threats that have the potential to become global emergencies. Its success will rely on the capacity and performance of national public health systems, anchored by strong national public health institutes (NPHIs). The new International Association of National Public Health Institutes aims to strengthen and invigorate existing NPHIs, to create new NPHIs where none exist, and to provide funded grants to support NPHI development priorities.

In the wake of the 2003 outbreak of severe acute respiratory syndrome (SARS), preparedness for public health emergencies was propelled into worldwide consciousness. The appearance and rapid international spread of SARS demonstrated to all—including global leaders, ministers of health, prime ministers, and heads of state—how an infectious disease can rapidly cross borders and deliver health threats and economic blows on an unimaginable scale (*1*,*2*). Since then, the entrenchment of highly pathogenic avian influenza virus (H5N1) in poultry flocks of Asian countries, and the spread of the virus across Europe and into Africa, has put the world on high alert for an influenza pandemic and affirmed the urgency of strengthening public health systems and capacity worldwide (*3*,*4*).

Compounding the challenges of threats to public health security from new and reemerging infectious diseases and the concerns about intentional dissemination of chemical or biological substances are the challenges of ensuring individual health security. These latter challenges include the unfinished agenda of broadening access to the drugs, vaccines, and other interventions needed to control endemic diseases such as malaria, acute lower respiratory tract infections, diarrheal diseases, measles, and tuberculosis, as well as to address the ongoing problems of HIV/AIDS, neglected tropical diseases, humanitarian emergencies, and global environmental changes.

The scale, range, and complexity of these modern challenges to health security call for new approaches of comparable dimension and strength. Protecting the world from transnational health threats demands a global public health perspective and investment in global public health infrastructure. The theme of this year’s World Health Day and the World Health Report 2007 is “Global public health security—the need to reduce the vulnerability of people around the world to new, acute, or rapidly spreading risks to health, particularly those that cross international borders” (*5*). With a call to all nations to “invest in health, and build a safer future,” the World Health Organization (WHO) emphasizes the need for collaboration among nations to increase our collective capacity and infrastructure to respond to potential international health emergencies and other public health risks. As recent events have shown, global public health security is a complex, costly, and information-intense undertaking that requires strong national public health leadership and infrastructure, cross-border collaboration, capacity to identify problems rapidly and design real-time evidence-based solutions, well-trained and well-equipped workforces, well-functioning laboratories and service-delivery systems, capacity to sustain interventions, and ability to respond to unexpected events (*5*,*6*). Investment in these elements will strengthen not only global public health security but also the infrastructure needed to help broaden access to healthcare services and improve individual health outcomes, which would help break the cycles of poverty and political instability and thus contribute to national economic development and achievement of the Millennium Development Goals (*7*).

A key driver in the effort to strengthen global public health security is the framework of the newly revised International Health Regulations (IHR [2005]) (*8*), the legally binding global agreement designed to build and strengthen national alert and response systems. Unanimously agreed upon by the World Health Assembly on May 23, 2005, the regulations are the result of experience gained and lessons learned about global public health security over the past 30 years. This global legal framework constitutes a “major development in the use of international law for public health purposes” (*9*). It includes a commitment from WHO and from each of its 193 member states to improve capacity for disease prevention, detection, and response and provides ground rules to address national public health threats that have the potential to become global emergencies. The adoption of the new regulations ended a 10-year process of revision, stimulated by the pneumonic plague outbreak in India in 1994 (*10*) and the Ebola hemorrhagic fever outbreak in the former Zaire in 1995 (*11*). The revised regulations have now entered into force for all WHO member states.

## New Times, New Requirements

The revised regulations reflect a growing understanding that the best way to prevent the global spread of diseases is to detect and contain them while they are still local. WHO member states have obligations to rapidly assess and alert the global community about potential disease threats as well as to prevent and control the spread of disease inside and beyond their borders. Compared with the previous regulations, adopted in 1969 (*12*), IHR (2005) expands the scope of internationally reportable diseases and events, provides criteria for identifying novel epidemic events, and specifies conditions for involvement of the international community in outbreak responses. The revision includes the following 5 substantive changes.

### Expanded Scope

The previous regulations applied to only 3 infectious diseases: cholera, plague, and yellow fever. IHR (2005) reflects shifting concepts about disease control, shaped by recent and impending disease threats and the experiences of the past 2 decades in detecting and responding to disease outbreaks. The emergence and reemergence of a cascade of infectious diseases fueled by globalization and international travel (*13*), the threat of biological terrorism, and novel environmental threats (*14*) have spotlighted the need for heightened vigilance and increased capacity to recognize and manage public health risks and emergencies. The appearance and rapid international spread of SARS and the pandemic potential of circulating avian influenza (H5N1) strains—with their combined health and economic effects—confirmed the inapplicability of the 1969 IHR to most emerging and reemerging infectious diseases.

The revised regulations replace the previous disease-specific framework with one built on timely notification of all events that might constitute a public health emergency of international concern, taking into account the context in which an event occurs (*15*). The advantage of this approach is its applicability to existing threats as well as to those that are new and unforeseen. The regulations also recognize the existence of threats to public health outside the infectious disease context, such as those associated with natural disasters, industrial or chemical accidents, and other environmental changes, which might cross international borders.

### Decision Instrument and Notification

Expanding the scope of the IHR beyond reporting of 3 diseases to reporting of any public health emergency of international concern required an algorithm to assist in identification of such events. The resulting decision instrument (see [*8*]*,* Annex 2, p. 43) identifies a limited set of criteria for use by member states for fulfilling the obligation to determine whether an event occurring within their territory might constitute a public health emergency of international concern and therefore require formal notification to WHO within 24 hours of assessment. Essentially, the events that must be reported are those that fulfill at least 2 of the following criteria:

- Is the public health impact of the event serious?
- Is the event unusual or unexpected?
- Is there a significant risk of international spread?
- Is there a significant risk of international trade or travel restriction?

To facilitate the use of the decision instrument, which requires some judgment to answer each of the questions, Annex 2 of the Regulations provides specific examples of events that might constitute a public health emergency of international concern. In addition to this broad scope for notification, IHR (2005) includes a list of diseases for which a single case must be reported to WHO immediately, regardless of the context in which the disease occurs. This list includes smallpox, poliomyelitis due to wild-type poliovirus, human influenza caused by a new subtype, or SARS. In addition, an event involving certain other diseases (e.g., cholera, pneumonic plague, yellow fever, viral hemorrhagic fevers) calls for a careful evaluation using the decision instrument to determine whether notification is indicated. The need for recognition of specific diseases requires adequate diagnostic laboratory capacity.

After an event is reported, only the Director General of WHO can determine whether the event formally constitutes a public health emergency of international concern. However, the Director General shall first consult with the affected state party and hear the view of the emergency committee. This committee, composed of experts from the newly established IHR roster of experts, is specifically set up to review a reported event and provide advice to the Director General on whether an event constitutes a public health emergency of international concern and whether a temporary recommendation must be issued. On request, WHO will be able to provide technical support to affected countries, including the mobilization of the Global Outbreak Alert and Response Network.

### Focal and Contact Points

A third innovation under IHR (2005) is the requirement for member states to designate “national IHR focal points” as the operational link for notification and reporting to WHO and for WHO to name corresponding “IHR contact points.” Effective communication between these 2 organizational entities will be central to the rapid management of a possible public health emergency of international concern. IHR focal points, or their designees, are required by IHR (2005) to be accessible at all times.

### National Core Surveillance and Response Capacities

Experiences during the past several years have shown that public health emergencies expose the weaknesses and vulnerabilities of national and subnational public health infrastructure. The fourth change calls for member states to develop, strengthen, and maintain core capacities to 1) detect, assess, notify, and report disease events, and 2) respond promptly and effectively to public health risks and public health emergencies of international concern. State parties are required to complete a capacity assessment within 2 years of the revised IHR entering into force and, after this assessment, to develop public health infrastructure and human resources that ensure full compliance within 5 years of the IHR entering into force. This assessment must lead to the development of national action plans to meet the core capacity requirements that Annex 1 of the Regulations specifies for different levels (i.e., local community or primary, intermediate, and national public health response) as well as designated airports, ports, and ground crossings. For these national points of entry, IHR (2005) also introduces special provisions for travelers, including the obligation to treat them with respect for their dignity, human rights, and fundamental freedom.

### WHO Support

WHO is required to assist all member states in fulfilling the new obligations. On request, WHO will collaborate with countries to evaluate their public health capacities and facilitate technical cooperation, logistical support, and mobilization of financial resources for strengthening capacity in surveillance and response. Countries will build on existing national or regional strategies such as the Asia Pacific Strategy for Emerging Diseases in WHO’s Southeast Asia and Western Pacific Regions (*16*) and the Integrated Disease Surveillance and Response strategy in the African Region (*17*). In many countries, national action plans can also build on the influenza pandemic preparedness plans developed with WHO’s guidance. Specific WHO guidelines and initiatives, particularly in the areas of external quality assessment for laboratories, data gathering and analysis at the health district level, and the central and coordination functions of national public health institutes, are being developed. WHO’s Lyon Office for National Epidemic Preparedness and Response is specifically dedicated to supporting countries in meeting the core national capacity requirements of IHR (2005).

Under IHR (2005), new powers for WHO include an information-gathering responsibility that is not limited solely to official state notifications or consultations but covers all available scientific evidence and other relevant information. WHO can consult nonofficial reports and require countries to collaborate with a request for verification. WHO is also empowered to recommend and coordinate measures that will help contain the international spread of disease, including public health actions at ports, airports, and land borders, and on means of transportation that involve international travel.

## Critical Role of National Public Health Institutes

Because weak national public health capabilities undermine efforts to strengthen global public health security, IHR (2005) imposes substantial responsibilities on countries to improve public health capacity and infrastructure. However, despite the broad new goals included in IHR (2005), improvements in global public health security will depend on what member states are actually able to do. Success will rely on the capacity and performance of national public health systems (*15*), anchored by strong national public health institutes (NPHIs). Low-resource countries, which are particularly vulnerable to emerging threats, will be particularly challenged by the IHR (2005) requirements and the need to ensure an appropriate and coordinated public health response to health emergencies.

Many countries have been well served by centralizing their public health expertise and activities within 1 institution or network of institutions that provides leadership and coordination for public health ( *18;* unpub. data). Examples include the US Centers for Disease Control and Prevention, the National Public Health Institute of Finland, and the Chinese Center for Disease Control and Prevention. These NPHIs are usually governmental or quasi-governmental agencies with a central focus and organizational structure that allow coordination of national public health service delivery and ensure a country’s ability to detect, investigate, and respond to public health emergencies. The core functions of an NPHI have been defined (unpub. data) and include evaluation and analysis of health status; public health surveillance, problem investigation, and control of risks and threats to public health; and public health research.

Given the scope and range of their activities, NPHIs are a vital asset to health development and security and will have a critically important role in implementing IHR (2005), whether as national focal points or as operational partners in fulfilling the requirements of the regulations. Unfortunately, however, many countries still either have no NPHIs or have institutes with severely limited capacities and capabilities relative to the need. Even in countries with strong NPHIs, unpredictable and rapidly evolving health threats can quickly overwhelm capacity and inhibit a timely and complete response.

A new organization, the International Association of National Public Health Institutes (IANPHI, www.ianphi.org), was created to address these gaps through the enhancement and proliferation of NPHIs throughout the world. Founded in 2006 by 39 NPHI directors who recognized the importance of strong national public health capacity and the mutual benefits of shared information, experience, and expertise, IANPHI aims to be a catalyst for sustained improvements in public health capacity and infrastructure globally. With the partnership of WHO and funding first from the Rockefeller Foundation and now from the Bill and Melinda Gates Foundation, the organization focuses on strengthening public health capacity in low-resource countries by strengthening NPHIs and on providing tools and a context that will support all NPHIs. IANPHI is also a professional association for NPHI directors; it fosters leadership development and advocacy for public health and collaborates with WHO.

Since early 2006, the founding members have continued to expand the network and put their shared vision into practice. IANPHI is managed by an executive board and a secretariat located both in Finland and in the United States. With nearly 50 current members and an ambitious agenda for collaboration, service, and growth, IANPHI is committed to a vision of a robust and fully integrated global network of NPHIs equipped to address critical public health challenges. Its mission is to strengthen and reinvigorate existing NPHIs, create new NPHIs where none exist, and provide funded grants to support NPHI capacity development priorities.

IANPHI achieves its service mission through a 3-part approach of advocacy, technical assistance, and linkages. IANPHI advocates for NPHI development and proliferation through partnerships with key global health organizations, such as WHO. Through these partnerships, IANPHI ensures that NPHIs are considered in major global public health initiatives and that public health and the work of NPHIs are included in efforts to strengthen health systems.

Assistance to NPHIs in low-resource countries is provided through 3 grant programs. A short-term technical assistance program helps countries quickly resolve priority gaps in NPHI capability and infrastructure. A medium-term capacity-building program helps NPHIs address high-priority needs for up to 3 years. IANPHI’s long-term grant program, the most intensive of the assistance efforts, will help create NPHIs in low-resource countries that currently lack a central public health focus. With funding from a $20 million grant from the Bill and Melinda Gates Foundation, the organization is committed to implementing 60 NPHI development projects by 2011.

As of June 2007, IANPHI had awarded technical assistance grants to public health institutes in 5 nations. The new awards include 3 short-term grants to NPHIs in Thailand, Uganda, and Iran to support training and infrastructure development. A medium-term grant to the Nigerian Institute of Medical Research will support sustainable improvements in disease surveillance, outbreak investigation, and emergency preparedness and strengthen linkages with other groups working to advance public health in the country; special focus will be on public health laboratory capacity building and integration of surveillance, epidemiology, and laboratory programs. Colombia’s Institutos Nacional de Salud was awarded a medium-term grant to establish a pilot chronic disease study site to generate, collect, and disseminate chronic disease data by using multiple mechanisms. The activities are designed to yield a sustainable network of surveillance and research sites to guide national-level public health decision making.

The cornerstone of the IANPHI approach is a peer-assistance model for NPHI strengthening and enhancement, with an emphasis on countries without NPHIs or with NPHIs in their early stages. Experts from IANPHI member institutes provide technical assistance and project support targeted at critical NPHI needs. Teams are guided by the Framework for the Creation and Development of NPHIs (www.ianphi.org/?action=arkisto&RYHMA=47&ID=&valittu=8), a product of IANPHI in partnership with WHO. The Framework provides a working definition of an NPHI and suggests a process for creating or enhancing an institute. By defining the critical characteristics of an NPHI, IANPHI hopes to bring specificity to the organization’s vision, align efforts to assist low-resource countries in building NPHIs, and provide benchmarks and resources to help any country assess and improve the functioning of its NPHI. To that end, IANPHI has also developed an NPHI toolkit (www.sph.emory.edu/IANPHI), which provides access to a variety of Web-based information resources for countries, NPHIs, and IANPHI peer-assistance teams to use as they work to assess, develop, and improve NPHIs and build public health capacity around the world.

Through strategies to define and develop core public health functions and to share expertise, IANPHI helps NPHIs sharpen their focus and raise standards of performance. IANPHI also links NPHIs through annual meetings, regional events, leadership development activities, research seed grants, and communication outlets including a website, newsletter, and listserv. By fostering an international community of public health leadership, IANPHI helps NPHIs gain the benefits of shared information, experience, and expertise to address public health threats and opportunities. Through its grant program and other activities, IANPHI aims to help national governments develop organizational infrastructures to devise and implement comprehensive public health priorities, meet global public health goals, develop workforce capacity, effectively absorb donor funds, address emerging threats, and improve the health of their populations (*18*).

## Conclusions

In today’s global environment, every country confronts similar challenges in keeping its population healthy and preventing the cross-border spread of disease. SARS demonstrated this dramatically in 2003, and the ongoing challenges posed by avian influenza have focused attention on the need for global pandemic influenza preparedness. Polio has reemerged in countries that had virtually eradicated it, while HIV/AIDS and other diseases continue to threaten the stability of communities around the world. Recent examples of emerging and reemerging diseases of global significance are the resurgence of dengue in tropical and subtropical areas of the world; the spread and establishment of Japanese encephalitis and West Nile viruses in new habitats and environments; and the reoccurrence and spread of chikungunya virus in India, East Africa, and several Indian Ocean islands (*19**,**20*). As life expectancy increases worldwide, issues related to noncommunicable conditions are also becoming increasingly common to all. By working within the collaborative framework provided by IHR (2005), countries can benefit through improved national and international surveillance; improved systems for rapid detection of and response to public health emergencies; standardized rules for evaluation, control, and resolution of urgent events; and mechanisms to increase national and local public health security.

Nonetheless, the success of IHR (2005) and other global public health initiatives such as the Millennium Development Goals depends on strong national public health systems with competent, well-trained staff and well-equipped facilities. By targeting the core of public health systems, especially in low-resource countries that currently lag behind in public health capacity and infrastructure, IANPHI will play a key role in improving the capacity of countries to effectively detect, investigate, and respond to public health emergencies. The result will be better control of endemic diseases such as HIV/AIDS, acute lower respiratory tract infections, diarrheal diseases, measles, tuberculosis, malaria, and the neglected tropical diseases. These efforts will strengthen the practice of public health worldwide, yield global public health benefits of disease control and prevention, and ultimately accelerate social and economic development in the poorest countries of the world and progress toward achieving the Millenium Development Goals.
